# Supplementary material for: The “Narratives” fMRI dataset for evaluating models of naturalistic language comprehension
Source: Sci Data. 2021 Sep 28;8:250. doi: 10.1038/s41597-021-01033-3 (PMC8479122; doi:10.1038/s41597-021-01033-3)
Supplement: Supplementary file 1 — Supplementary Information [file 41597_2021_1033_MOESM1_ESM.docx]

**Supplementary information**

| **Script** | **Description** |
| --- | --- |
| compile_metadata.py | Compile dictionaries containing metadata and filenames for each subject (subject_meta.json) and task (task_meta.json). |
| brain_masks.py | Create brain masks for data in MNI space and fsaverage6 space. |
| roi_masks.py | Create early auditory cortex ROI masks based on multimodal cortical parcellation. |
| run_pydeface.py | Run PyDeface to anonymize (de-face) each subject’s anatomical image(s). |
| run_mriqc.sh | Run participant-level MRIQC on BIDS-formatted data for one subject. |
| slurm_mriqc.sh | Submit Slurm job array to run MRIQC on many subjects in parallel. |
| run_mriqc_group.sh | Run group-level MRIQC to summarize participant-level MRIQC outputs. |
| run_fmriprep.sh | Run fMRIPrep on BIDS-formatted data for one subject. |
| slurm_fmriprep.sh | Submit Slurm job array to run fMRIPrep on many subjects in parallel. |
| run_smoothing.py | Run spatial smoothing using AFNI’s 3dBlurToFWHM and SurfSmooth for one subject. |
| slurm_smoothing.sh | Submit Slurm job array to run spatial smoothing on many subjects in parallel. |
| extract_confounds.py | Extract confound variables from fMRIPrep outputs for use with AFNI’s 3dTproject. |
| run_regression.py | Run confound regression using AFNI’s 3dTproject for one subject. |
| slurm_regression.py | Submit Slurm job array to run confound regression on many subjects in parallel. |
| run_isc.py | Run whole-brain vertex-wise leave-one-out intersubject correlation (ISC) analysis on smoothed surface data across all subjects in each story. |
| roi_average.py | Average non-smoothed time series across vertices within early auditory cortex ROI. |
| roi_isc.py | Compute leave-one-out ISC for early auditory cortex ROI. |
| roi_lags.py | Compute ISC for early auditory cortex ROI at lags ranging from -30 to +30 TRs. |
| exclude_scans.py | Compile dictionary (scan_exclude.json) for excluding scans based on ISC in early auditory cortex ROI with exclude_scan function. |
| get_demog.py | Summarize demographic information (age, sex) based on participants.tsv file |
| get_words.py | Summarize the number of words (including missing and unknown) across all stimuli. |
| get_tsnr.py | Estimate tSNR using AFNI’s 3dTstat and compute median tSNR across all scans. |
| get_fwhm.py | Estimate intrinsic smoothness using AFNI’s 3dFWHMx for one subject. |
| slurm_fwhm.py | Submit Slurm job array to run smoothness estimation on many subjects in parallel. |
| plot_stim.py | Plot audio waveform for Pie Man stimulus with example voxel time series (Figure 1). |
| plot_qc.py | Plot tSNR and FD from MRIQC, as well as intrinsic smoothness (Figure 2). |
| plot_isc.py | Plot ISC and lagged ISC for the early auditory cortex ROI (Figure 3). |
| gifti_io.py | Helper functions for reading and writing GIfTI surface files in Python. |

**Supplementary Table 1.** Scripts used to process the “Narratives” data with brief descriptions. Scripts are listed roughly in order of execution. All scripts are located in the code/ directory of the BIDS dataset and in the accompanying GitHub repository (<https://github.com/snastase/narratives>).
